# R2R3-MYB Transcription Factor NtMYB330 Regulates Proanthocyanidin Biosynthesis and Seed Germination in Tobacco (*Nicotiana tabacum* L.)

**DOI:** 10.3389/fpls.2021.819247

**Published:** 2022-01-17

**Authors:** Lu Zhao, Zhongbang Song, Bingwu Wang, Yulong Gao, Junli Shi, Xueyi Sui, Xuejun Chen, Yihan Zhang, Yongping Li

**Affiliations:** ^1^Key Laboratory of Tobacco Biotechnological Breeding, Yunnan Academy of Tobacco Agricultural Sciences, Kunming, China; ^2^National Tobacco Genetic Engineering Research Center, Yunnan Academy of Tobacco Agricultural Sciences, Kunming, China

**Keywords:** proanthocyanidin, R2R3-MYB transcription factor, MYB-bHLH-WDR complex, seed germination, ABA/GA signaling

## Abstract

Proanthocyanidins (PAs) are important phenolic compounds and PA biosynthesis is regulated by a ternary MBW complex consisting of a R2R3-MYB regulator, a bHLH factor and a WDR protein. In this study, a tobacco R2R3-MYB factor NtMYB330 was characterized as the PA-specific regulator in which the PA biosynthesis was promoted in the flowers of *NtMYB330*-overexpressing lines while decreased in the flowers of *ntmyb330* mutants. NtMYB330 can interact with flavonoid-related bHLH partner NtAn1b and WDR protein NtAn11-1, and the NtMYB330-NtAn1b complex is required to achieve strong transcriptional activation of the PA-related structural genes *NtDFR1*, *NtANS1*, *NtLAR1* and *NtANR1*. Our data reveal that NtMYB330 regulates PA biosynthesis in seeds and affects seed germination, in which *NtMYB330*-overexpressing lines showed higher PA accumulations in seed coats and inhibited germination, while *ntmyb330* mutants had reduced seed coat PAs and improved germination. NtMYB330 affects seed germination possibly through two mechanisms: modulating seed coat PAs to affect coat-imposed dormancy. In addition, NtMYB330 regulates the expressions of abscisic acid (ABA) and gibberellin acid (GA) signaling-related genes, affecting ABA-GA crosstalk and seed germination. This study reveals that NtMYB330 specifically regulates PA biosynthesis via formation of the MBW complex in tobacco flowers and affects germination through adjustment of PA concentrations and ABA/GA signaling in tobacco seeds.

## Introduction

Proanthocyanidins (PAs), also known as condensed tannins, are a class of polyphenols found in a wide variety of plant species (Dixon et al., 2005). Its biosynthesis shares early biosynthetic genes (EBGs) of flavonoid pathway including chalcone synthase (*CHS*), chalcone isomerase (*CHI*) and flavanone 3-hydroxylase (*F3H*) with flavonols and anthocyanins (Appelhagen et al., 2011). PA and anthocyanin biosynthetic pathways share late biosynthetic genes (LBGs) such as dihydroflavonol 4-reductase (*DFR*) and leucoanthocyanidin dioxygenase (LDOX/ANS), leading to the productions of leucoanthocyanidins and anthocyanidins, respectively. These two substrates are converted to (+)-catechin and (−)-epicatechin, catalyzed by leucoanthocyanidin reductase (LAR) and anthocyanidin reductase (ANR), respectively (Bogs et al., 2005). PA oligomers are then formed by sequentially adding the intermediates derived from flavan-3,4-diols to catechin or epicatechin (Abrahams et al., 2002).

R2R3-myeloblastosis (MYB) transcription factors are involved in regulating the biosynthesis of different flavonoid branches. For the regulation of PA and anthocyanin biosynthesis, MYB factors interact with basic helix-loop-helix (bHLH) and WD-repeat (WDR) co-activators to form a ternary complex to control gene expression (Ramsay and Glover, 2005). The N-termini of these bHLH-interacting R2R3-MYBs are highly conservative, containing a bHLH interaction motif [D/E]Lx_2_[R/K]x_3_Lx_6_Lx_3_R in R3 repeat of R2R3-MYB domain, while the C-termini are variable, containing transcriptional activation or repression domains which contribute to the biological specificity (Feller et al., 2011). Based on the evolutional analysis of R2R3 region, PA- and anthocyanin-regulating MYB factors are likely to evolve from a common ancestral MYB precursor, in which PA-regulating MYB factors have evolved two separate PA subclades as indicated by PA-clade 1 and PA-clade 2, while one branching of anthocyanin-regulating MYB factors evolved after the separation of two PA subclades (Akagi et al., 2010; Heppel et al., 2013). PA-specific MYB regulators placed in two subclades are distinct in gene structures, conserved C-terminal motifs and targeted *cis*-motifs in promoter regions. Genes in PA-clade 1 have one intron while those in PA-clade 2 have two introns. For the conserved motifs in C-terminal domain, PA-clade 1 regulators harbor K[I/V]x2PKPx1Rx2S[I/L] motif (Zhu et al., 2015; Passeri et al., 2017), while PA-clade 2 regulators contain VI[R/P]TKAx_1_RC[S/T] motif (Nesi et al., 2001; Terrier et al., 2009). Moreover, PA-clade 1 regulators recognize the MYBCORE element, while PA-clade 2 regulators mainly target AC elements in the promoter regions of PA pathway genes (Hichri et al., 2011b).

In herbaceous plants, PAs are commonly found in epidermal layer of seed coats, protecting embryo and endosperm (Lepiniec et al., 2006). PAs accumulate in the vacuole within the endothelial cells and the oxidized PA polymers interact with cell wall components by making cross-links with proteins and polysaccharides (Marles et al., 2003), producing brown pigments during seed desiccation (Baxter et al., 2005; Lepiniec et al., 2006). Seed coat PAs are positively associated with the dormant state and inhibition of germination by PAs has been demonstrated in a number of plants. In tomato, overexpression of *SlAn11* gene resulted in increased endogenous PA levels along with arrested seed germination compared to the wild-type (Gao et al., 2018). Low temperature, as an important environmental cue triggering seed dormancy during seed set in Arabidopsis, can cause higher accumulations of seed coat PAs. While *transparent testa* (*tt*) mutant with low PA concentrations had increased germination phenotype after maturation at low temperatures (MacGregor et al., 2015). The inhibitory effect of PAs on seed germination is presumably achieved through promoting the biosynthesis of abscisic acid (ABA), since PA-deficient mutants with reduced seed dormancy also exhibit lower ABA levels during seed germination (Jia L. et al., 2012).

**FIGURE 1:**
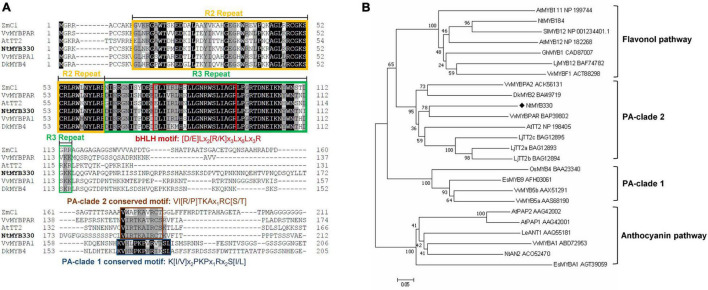
Multiple alignment and phylogenetic analysis of selected R2R3-MYB proteins. **(A)** The deduced amino acid sequence of NtMYB330 was aligned with R2R3-MYB homologous ZmC1 (AAA33482) from *Zea mays*, VvMYBPAR (BAP39802) and VvMYBPA1 (CAJ90831) from *Vitis vinifera*, AtTT2 (NP198405) from *Arabidopsis thaliana*, DkMYB4 (BAI49721) from *Diospyros kaki*. The R2 and R3 repeats are boxed in orange and green, respectively. The bHLH motif [D/E]Lx_2_[R/K]x_3_Lx_6_Lx_3_R, PA-clade 1 conserved motif K[I/V]x_2_PKPx_1_Rx_2_S[I/L] and PA-clade 2 conserved motif VI[R/P]TKAx_1_RC[S/T] are indicated in red, dark blue, and brown boxes, respectively. **(B)** The phylogenetic tree of NtMYB330 along with selected R2R3-MYBs was constructed using neighbor-joining method. Scale bar represents 0.05 substitutions per site and numbers next to nodes are bootstrap values from 1000 replicates. GenBank accession numbers are indicated next to the protein name.

As flavonoids are related to stress resistance, plant growth and development, crop quality, and so on, flavonoid biosynthesis in higher plants has been extensively studied. However, the studies in tobacco mainly focus on the regulation of flavonol and anthocyanin biosynthesis, the regulation of PA pathway remains unclear. As a model plant, tobacco is chiefly used as a platform for functional verification of exogenous PA-related regulators. Therefore, it is necessary to study the underlying mechanism of PA regulation in tobacco and analyze the similarities and differences in PA regulations between tobacco and other higher plants. In previous study, several MYB regulators had been identified, which were co-expressed with the structural genes in flavonoid pathway (Jiao et al., 2020). Among them, NtMYB330 was found to fall into PA-clade 2 (Figure 1B) and was selected for functional analysis in present study. Our results reveal that NtMYB330 regulates PA biosynthesis in tobacco flowers, and the regulatory function of NtMYB330 relies on the interaction with a bHLH partner. In addition, NtMYB330 can regulate seed coat PAs and affect seed germination, possibly by modulating the permeability and thickness of seed coats, and/or affecting the ABA-GA crosstalk during seed germination.

## Materials and Methods

### Plant Materials

Seeds of *Nicotiana tabacum* L. cv. Yunyan 87 were germinated in Murashige and Skoog (MS) medium and the seedlings were transferred into magenta box for further development. To analyze tissue-specific expressions of *NtMYB330*, different tobacco tissues were harvested from a flowering plant. Wild-type and T_2_ transgenic tobaccos were grown in a greenhouse under natural light conditions at 25°C.

### Sequence Analysis of NtMYB330

The full-length coding sequence of *NtMYB330* (Nitab4.5_0000189g0020) was obtained from Sol Genomics Network^1^. The deduced amino acid sequences of NtMYB330 and homologous MYB regulators identified in NCBI through BLASTp search were aligned in MEGA 7.0 package using ClustalW program. The phylogenetic tree was constructed and visualized using neighbor-joining method by MEGA 7.0 package. The statistical reliability of individual nodes of the tree was assessed by bootstrap analysis with 1,000 replications.

### Plasmid Construction and Tobacco Transformation

For construction of the overexpression vector, CDS fragment of *NtMYB330* was amplified from cDNAs by PCR using gene specific primers (Supplementary Table 1). The PCR conditions were as follows: 30 s pre-denaturation at 98°C, followed by 7 s denaturation at 98°C, 30 s annealing at 62°C, and 45 s elongation at 72°C for 35 cycles with a final extension at 72°C for 7 min. The 1,164-bp amplicons were then ligated to the binary vector pK2GW7 (Karimi et al., 2002) resulting in pK2GW7-*NtMYB330*. For construction of the Cas9/sgRNA vector, sgRNA sequence containing 20 nucleotides (TTGTTTAATCCTTCTTTAGA) located in the first exon of *NtMYB330* gene was generated and then subcloned into the CRISPR/Cas9-based binary vector (Xing et al., 2014) to form the transformation vector pCas9-*NtMYB330*.

Transgenic tobacco plants were generated though *Agrobacterium tumefaciens*-mediated leaf disk transformation (Palmgren et al., 1993). Empty vector (pK2GW7) was transformed to generate tobacco plants as vector control lines. Positive transgenic plants, selected with kanamycin resistance were confirmed by PCR using nptII_F/R primers (Supplementary Table 1). The PCR program was pre-incubation at 98°C for 30 s, 30 cycles of amplification at 98°C for 7 s, 58°C for 30 s, and 72°C for 30 s, followed by a final extension at 72°C for 5 min. PCR-validated transgenic plants were self-pollinated twice to generate T_2_ transgenic progeny. For the *ntmyb330* mutants, T_1_ seeds were generated by self-pollinating independent T_0_ plants with InDels introduced by CRISPR/Cas9 genome editing at the target site. T_1_ plants were screened for the presence of transgene event by amplifying hygromycin resistance gene using hpt_F/R primers (Supplementary Table 1). The PCR program was pre-incubation at 98°C for 30 s, 30 cycles of amplification at 98°C for 7 s, 65°C for 30 s, and 72°C for 30 s, followed by a final extension at 72°C for 5 min. T_1_ transgenic-free plants harboring homozygous mutations in *NtMYB330* were self-crossed to generate T_2_ seeds for subsequent analyses.

### Gene Expression Analysis

Total RNA was isolated using the RNeasy kits (Qiagen) following the manufacturer’s instructions and then treated by RNase-free DNAse I (New England Biolabs) to remove residue genomic DNA. First-strand cDNA was generated using PrimeScript RT reagent (Takara). Quantitative real-time PCR (qPCR) was performed using a LightCycler thermocycler (LightCycler^®^ 480 Instrument II, Roche Diagnostics, Rotkreuz, Switzerland) with 1X LightCycler^®^ 480 SYBR Green I Master (Roche Applied Science, Manheim, Germany) as the fluorochrome. The tobacco *actin* gene was used as an internal reference control. The PCR program was 30 s pre-denaturation at 95°C, followed by 30 s denaturation at 95°C, 20 s annealing at optimal temperature of each primer pair, and 20 s elongation at 72°C for 40 cycles. The specificity of amplification was analyzed by the melting curve method. Relative quantification of gene expression was carried out using the 2^–△△Ct^ method. All the gene-specific primers for qPCR are listed in Supplementary Table 1.

### Proanthocyanidin Measurement

Proanthocyanidins presented in tobacco flower petals and seed coats were visualized by staining with 4-dimethylaminocinnamaldehyde (DMACA) solution (0.3% DMACA in 6N HCl: 100% ethanol, 1:1) (Li et al., 1996) for 30 min. For DMACA staining, flower petals were collected during flowering stage and seeds were harvested at 18 days after pollination (Park et al., 2007). Following DMACA staining, flower petals and seeds were washed by 70% ethanol, then rinsed with distilled water for three times. The histochemical staining of seed coats was observed under the microscope.

For PA extraction, fresh tobacco flowers collected during flowering stage were ground in a mortar and pestle using liquid nitrogen and then were lyophilized. An aliquot (50 mg) of flower powder was suspended in 1 mL of 80% acetonitrile, ultrasonicated for 30 min, then centrifuged at 14,000 rpm for 10 min. Supernatants were passed through 0.22 μm membrane filter prior to injection on the UPLC-MS/MS system. Quantification of PAs was conducted in an Acquity UPLC system (Waters Corp., Milford, MA, United States) coupled with an AB Sciex QTrap 5500 triple-quadrupole mass spectrometer with electrospray ionization (ESI) (AB Sciex, Foster City, CA, United States). The UPLC system was equipped with a 100 mm × 2.1 mm i.d., 1.7 μm, Acquity UPLC BEH C18 Phenyl column (Waters, Ireland), an autosampler, a binary solvent manager and a diode array detector. The column temperature was set to 25°C and the injection volume was 2 μL. The mobile phase consisted of (A) acidified methanol (methanol contains 0.2% acetic acid, v/v) and (B) 0.2% acetic acid (v/v) at a flow rate of 0.35 mL/min. The elution profile was as follows (A): 0–1 min: 5% isocratic; 1–3 min: 5–25%; 3–5 min: 25% isocratic; 5–9 min: 25–80%; 9–11 min: 80% isocratic; 11–11.1 min: 80-5%; 11.1–13 min: 5% isocratic. MS data was collected continuously from 0 to 10 min, and analytes were detected as negative ions. The ESI source operation parameters were as follows: source temperature 600°C; ion spray voltage −4,000 V; ion source gas I, gas II and curtain gas were set at 60, 50, and 40 psi, respectively. Metabolites were quantitatively analyzed based on the multiple reaction monitoring (MRM) mode and the characteristic ions of catechin and epicatechin were screened through the mass spectrometer to obtain signal strengths (Supplementary Table 2). The corresponding contents of catechins and epicatechins in the flowers of different tobacco lines were shown as chromatographic peak area integrals (Supplementary Figure 7). Catechin and epicatechin were purchased from Shanghai Aladdin Bio-Chem Technology Co., Ltd (Shanghai, China).

### Subcellular Location of the GFP-NtMYB330 Fusion Protein

The pCAMBIA1300-*35S-GFP-NtMYB330* plasmid was introduced into leaves of *N. benthamiana* at 5-week-old by *Agrobacterium*-infiltration. pCAMBIA1300-*35S-N-GFP* was transfected as a positive control. Leaf samples were collected 72 h post transformation and used to capture the fluorescent signal using a confocal laser scanning microscope (Zeiss LSM510 Meta, Jena, Germany).

### Yeast-Two-Hybrid Screening

The full-length CDS of *NtAn11-1* and the MYB-interacting region (MIR) of NtAn1b*^aa1–195^* were PCR amplified and ligated to pGBKT7 (bait). To determine the threshold of 3-amino-1,2,4-triazole (3-AT) in inhibiting the background growth of baits, the bait vectors were transferred to yeast strain AH109 and grown on SD medium lacking tryptophan and histidine (SD/-Trp/-His) and supplemented with 3-AT at 0, 2.5, 5, 7.5, 10, or 15 mM, respectively. The background growths of BD-NtAn1b*^aa1–195^* and BD-NtAn11-1 were eliminated at 0 mM and 2.5 mM of 3-AT, respectively. The full-length CDS of *NtMYB330* were ligated to pGADT7 vector (prey) and the yeast strains harboring prey vectors were examined for toxicity on SD medium lacking leucine. The pAD-*NtMYB330* and pBD-*NtAn1b^aa1–195^*/*NtAn11-1* plasmids were co-transformed into yeast strain AH109 (Clontech, United States) according to manufacturer’s instructions. Transformed colonies were selected on SD medium lacking leucine and tryptophan (SD/-Leu/-Trp). Colonies from double selection plates (SD/-Leu/-Trp) were then screened for growth on the selective medium lacking leucine, tryptophan and histidine (SD/-Leu/-Trp/-His) and supplemented with 0 mM or 2.5 mM of 3-AT, and the SD/-Leu/-Trp/-His/-Ade medium containing X-α-Gal.

### Bimolecular Fluorescence Complementation Assay

The full-length cDNA of *NtMYB330* was cloned into pCAMBIA1300-NYFP as C-terminal fusions with the split YFP fragments (*pNYFP-NtMYB330*). The full-length CDS of *NtAn11-1* (aa 1-343) and the MIR of *NtAn1b* (aa 1-195) were cloned into pCAMBIA1300-CYFP as N-terminal fusions with the split YFP fragments (*NtAn11-1-pYFPC* and *NtAn1b_MIR-pYFPC*, respectively). All these plasmids were transformed into *Agrobacterium* strain EHA105 and then used to co-infect the leaves of *N. benthamiana* at 5-week-old. The green fluorescence signals were visualized using confocal laser scanning microscopy (Zeiss LSM510 Meta, Jena, Germany) at 72 h post infection.

### Dual Luciferase Assay

The transient expression assay was performed using the dual luciferase assay reagents (Promega, Madison, WI, United States). The reporter plasmids were generated by cloning the promoter fragments of *NtDFR1*, *NtANS1*, *NtLAR1*, and *NtANR1* into pGreenII 0800-LUC containing a firefly luciferase (LUC) CDS and a control promoter (CaMV35S) regulating expression of the *Renilla* (REN) luciferase reporter gene (Hellens et al., 2005). The effector plasmids were generated by cloning the full-length cDNA of *NtMYB330* or *NtAn1b* into pGreenII 62-SK. The reporter and effector plasmids were co-transformed into *N. benthamiana* leaf and the LUC activity relative to REN (LUC to REN ratio) was measured 72 h post infiltration using a Dual-Luciferase Reporter Assay System (Promega) with Modulus™ Luminometer (Promega, United States) following the manufacturers’ instructions.

### Seed Germination Assays

Seed germination assays were performed as previously described (Lv et al., 2021). Seeds with radicle breaking through the seed coats were counted as germinated. Germination percentages were measured for nine consecutive days. The experiments were repeated for three times.

## Results

### Isolation and Characterization of NtMYB330

**FIGURE 2:**
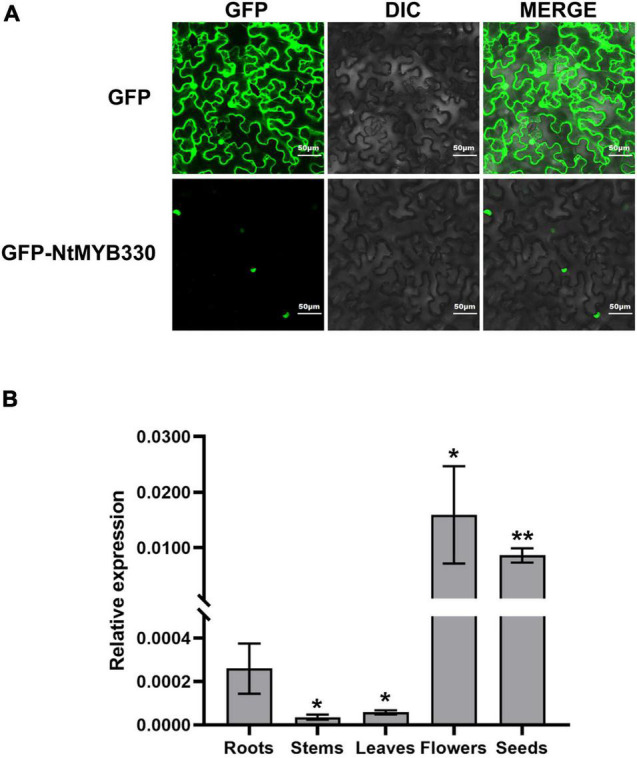
Subcellular localization of NtMYB330 protein and *NtMYB330* gene expression pattern. **(A)** Subcellular localization of GFP-NtMYB330 fusion protein and GFP control in *N. benthamiana* leaves transformed by *Agrobacterium*-infiltration. GFP, GFP fluorescence; DIC, differential interference contrast; MERGE, fluorescence and DIC merged images. Scale bar = 50 μm. **(B)** Relative expressions of *NtMYB330* in different tobacco tissues. Data are the mean of three replicates with error bars indicating ± SD. Asterisks indicate statistically significant differences from Roots according to paired *t*-test (*P < 0.05; **P < 0.01).

In previous study, the transcriptome analysis of pink-flowered tobacco cultivar Yunyan 87 vs. white-flowered Yunyan 87 mutant had found several genes encoding MYB and bHLH transcription factors downregulated along with the suppression of structural genes of flavonoid pathway in white flowered Yunyan 87 mutants (Jiao et al., 2020). In addition to the flavonoid-related MYB regulators discussed in Jiao et al. (2020), *NtMYB330* was also found downregulated in the flowers of white-flowered Yunyan 87 mutants and was selected to further characterize the gene function (Supplementary Figure 1). The deduced amino acid sequence of NtMYB330 was aligned to other MYB factors regulating flavonoid biosynthesis in dicots and monocots (Figure 1A). The protein N-terminal of NtMYB330 harbors R2R3 repeats corresponding to DNA-binding domain and [D/E]Lx_2_[R/K]x_3_Lx_6_Lx_3_R motif interacting with bHLH proteins, while the C-terminal region of NtMYB330 contains conserved motif VI[R/P]TKAx_1_RC[S/T]. The phylogenetic analysis showed that NtMYB330 was placed in the PA-clade 2 (Figure 1B), suggesting that NtMYB330 might be a PA-specific regulator in tobacco. To determine the subcellular localization of NtMYB330 protein, the green fluorescence protein (GFP) was fused to N terminus of NtMYB330 to make GFP-NtMYB330 fusion proteins. Transient expression in tobacco leaf cells showed that the fluorescence of fused GFP-NtMYB330 exclusively localized in the nucleus, while the fluorescence of control GFP protein was visible throughout the cells (Figure 2A). The transcript levels of *NtMYB330* in flowers and seeds were significantly higher than those in roots, while the transcript levels of *NtMYB330* in stems and leaves were significantly lower compared to those in roots (Figure 2B). These results suggested that NtMYB330 might be a PA-specific transcription factor and mainly functions in tobacco flowers and seeds.

### NtMYB330 Regulates Proanthocyanidin Biosynthesis in Tobacco Flowers

**FIGURE 3:**
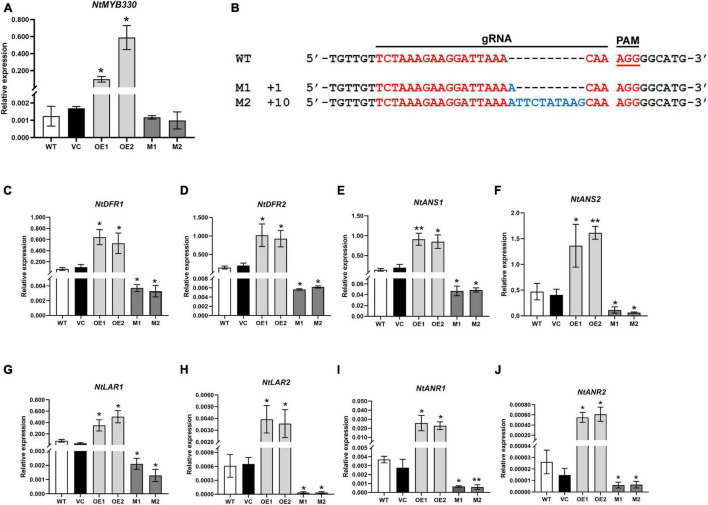
Quantitative transcript analyses of *NtMYB330* and LBGs in PA pathway in tobacco flowers. **(A)** Expressions of *NtMYB330* in the flowers of different tobacco lines. **(B)** Alignment of reads with CRISPR/Cas9-induced mutagenesis in *NtMYB330*. The wild-type sequence was shown at the top, and the two sequences on the bottom were reads of *ntmyb330* mutant lines (M1 and M2). The targeted sequence was shown in red, while mutations were shown in blue. gRNA sequences used for genome editing and PAM (protospacer adjacent motif) are indicated. The sizes of the InDels introduced are shown on the left. Analyses of expressions of **(C)** *NtDFR1*, **(D)** *NtDFR2*, **(E)** *NtANS1*, **(F)** *NtANS2*, **(G)** *NtLAR1*, **(H)** *NtLAR2*, **(I)** *NtANR1* and **(J)** *NtANR2* in the flower petals of wild-type (WT), vector control (VC), *NtMYB330-OE* lines (OE) and *ntmyb330* mutants (M). Data are the mean of three replicates with error bars indicating ± SD. Asterisks indicate statistically significant differences from WT according to paired *t*-test (*P < 0.05; **P < 0.01).

**FIGURE 4:**
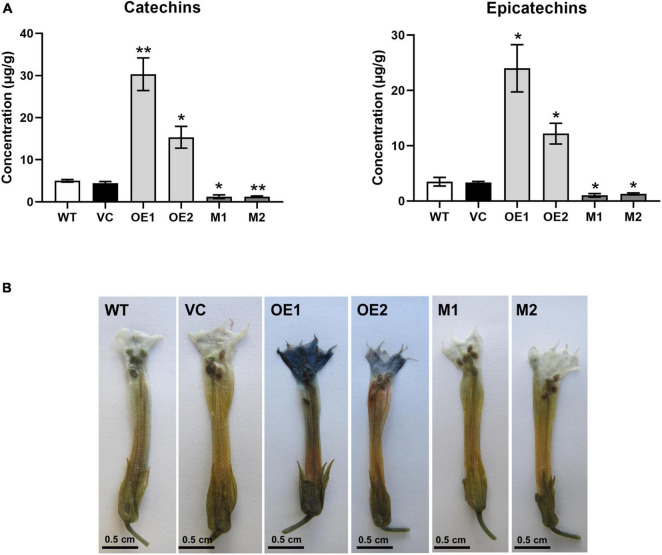
NtMYB330 regulates PA biosynthesis in tobacco flowers. **(A)** Concentrations of catechins and epicatechins in the flower petals of WT, VC, *NtMYB330-OE* and *ntmyb330* mutant plants. Data are the mean of three replicates with error bars indicating ± SD. Asterisks indicate statistically significant differences from WT according to paired *t*-test (*P < 0.05; **P < 0.01). **(B)** DMACA staining of PAs accumulated in the flower petals of WT, VC, *NtMYB330-OE* and *ntmyb330* mutant plants. Scale bar = 0.5 cm.

To characterize the function of NtMYB330 in regulating PA biosynthesis, *NtMYB330* overexpression (*NtMYB330-OE*) lines and knockout (*ntmyb330*) mutants were generated (Figures 3A,B). The transcript levels of *NtDFR1*, *NtDFR2*, *NtANS1*, *NtANS2*, *NtLAR1*, *NtLAR2*, *NtANR1*, and *NtANR2* were significantly increased in *NtMYB330-OE* flowers, while reduced in *ntmyb330* flowers compared with the wild-type control (Figure 3). Interestingly, the expressions of most EBGs (such as *NtCHIs*, *NtCHSs*, *NtF3Hs*, and *NtFLSs*) were not significantly altered in either *NtMYB330-OE* lines or *ntmyb330* mutants (Supplementary Figures 2A,B). NtAn1b and NtAn11-1 are flavonoid-related bHLH and WDR protein, respectively, isolated from tobacco flowers (Bai et al., 2011; Zhu et al., 2015). Overexpressing and/or knocking out *NtMYB330* gene did not dramatically change the expression levels of *NtAn1b* and *NtAn11-1* (Supplementary Figures 2C,D). The concentrations of catechins and epicatechins were significantly higher in *NtMYB330-OE* flowers, while *ntmyb330* mutant flowers accumulated significantly less of these compounds compared to the wild-type (Figure 4A). To visualize the PA accumulations in tobacco flowers, DMACA staining was performed, in which DMACA specifically binds to small PA oligomers and flavan-3-ols such as catechins, epicatechins, epigallocatechin and so on (Treutter, 1989). Flower petals of *NtMYB330-OE* lines turned dark blue after staining, whereas the petal colors of *ntmyb330* mutants, wild-type and vector controls were white (Figure 4B). Expression levels of PA-related genes and PA contents in the leaves of different lines were measured (Supplementary Figures 3, 4). However, no significant difference was observed, except for *NtDFR1*, which was upregulated in one of the *NtMYB330-OE* lines (Supplementary Figure 3A).

### NtMYB330 Interacts With Tobacco Basic Helix-Loop-Helix and WD-Repeat Protein to Form a Regulatory Complex

**FIGURE 5:**
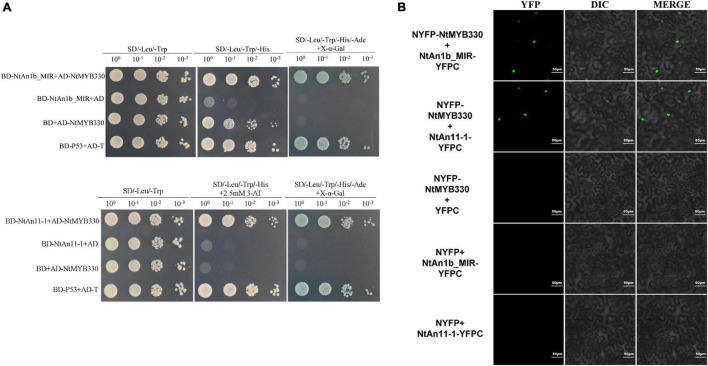
NtMYB330 interacts with NtAn1b_MIR and NtAn11-1. **(A)** Y2H assays reveal the interactions between NtMYB330 and NtAn1b_MIR, and between NtMYB330 and NtAn11-1. The coding sequence of the MYB-interacting region (MIR) of NtAn1b and the full-length coding sequence of NtAn11-1 were cloned into pGBKT7 to make fusion constructs BD-NtAn1b_MIR and BD-An11-1, respectively, and were co-transformed with pAD-NtMYB330. The interactions were indicated by yeast growth and X-α-Gal staining. Yeasts grown on medium with double (SD/-Leu/-Trp), triple (SD/-Leu/-Trp/-His) or quadruple (SD/-Leu/-Trp/-His/-Ade) selection were indicated. **(B)** BiFC assay shows the localization of NtMYB330–NtAn1b_MIR or NtMYB330-NtAn11-1 complex in epidermal cells of *N. benthamiana*. The full-length cDNA of *NtMYB330* was fused to N-terminal half of the yellow fluorescent protein (YFP), while cDNA of *NtAn1b_MIR* (aa 1–195) or the full-length cDNA of *NtAn11-1* was fused to C-terminal half of YFP. NYFP-NtMYB330 plus YFPC, NYFP plus NtAn1b_MIR-YFPC, and NYFP plus NtAn11-1-YFPC were used as the controls. Yellow signals indicate the positive interactions. Scale bar = 50 μm. Experiment was repeated three times, representative data from one experimental replicate presented here.

Previous study suggested that exogenous MYB factor can regulate PA biosynthesis in tobacco via formation of a MBW complex with tobacco flavonoid-related bHLH (NtAn1b) and WDR (NtAn11-1) proteins (Zhu et al., 2015). To test the binding affinity between NtMYB330 and flavonoid-related regulators, Y2H screenings were conducted. Yeast colonies from the combined transformations grew on triple (SD/-Leu/-Trp/-His) and quadruple (SD/-Leu/-Trp/-His/-Ade) selective media, suggesting that NtMYB330 can interact with NtAn11-1 protein and the MYB-interaction region (MIR) of NtAn1b (Figure 5A). By performing BiFC assays, we observed green fluorescence signals in the nuclei of tobacco cells co-expressing *NYFP-NtMYB330* and *NtAn1b_MIR-YFPC*, or co-expressing *NYFP-NtMYB330* and *NtAn11-1-YFPC* (Figure 5B). No green fluorescence was detected in the cells when *NYFP-NtMYB330*, *NtAn1b_MIR-YFPC*, or *NtAn11-1-YFPC* was co-transformed with the vector controls (C-terminal or N-terminal halves). These results further confirmed that NtMYB330 can interact with NtAn11-1 protein and the MIR of NtAn1b to form a regulatory complex *in vivo*.

### NtAn1b Is Essential for NtMYB330 to Regulate Proanthocyanidin-Related Genes

**FIGURE 6:**
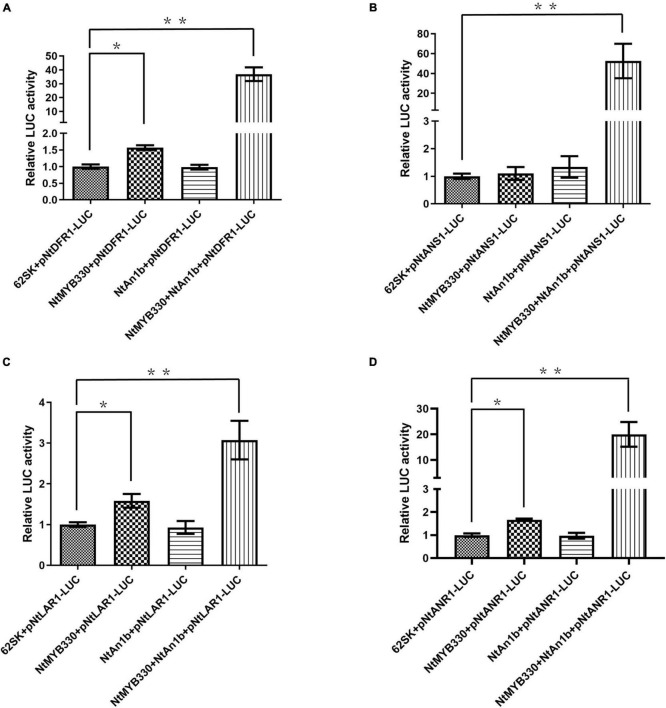
Transactivation assays of NtMYB330 and NtAn1b in *N. benthamiana* leaves. The reporter plasmids contain a firefly luciferase gene driven by the promoters of **(A)** *NtDFR1*, **(B)** *NtANS1*, **(C)** *NtLAR1*, or **(D)** *NtANR1*. The effector plasmids contain the full-length cDNA of either *NtMYB330* or *NtAn1b*, and were co-transformed, alone or in combination, with the reporter plasmid into the *N. benthamiana* leaf. A pGreenII 62-SK plasmid was used as the vector control (62SK). Results are presented as fold activation compared to the vector control. Error bars represent the mean ± SD of nine biological replicates. Asterisks indicate statistically significant differences from the vector control (*P < 0.05; **P < 0.01) using one-way ANOVA followed by Bonferroni test.

Previous study found that NtAn1b can regulate *ANR* expression only when interacting with the anthocyanin-regulating MYB but not the WDR protein (Tian et al., 2020). To determine whether NtAn1b is required for NtMYB330 in regulating PA-related genes, transactivation activities of NtMYB330 and NtAn1b were evaluated (Figure 6). Interestingly, co-expression of *ProDFR1*/*LAR1*/*ANR1::LUC* and NtMYB330 resulted in much stronger luminescence intensity compared to the reporter only controls (Figures 6A,C,D). When NtMYB330 and NtAn1b were co-infiltrated along with *ProDFR1*/*ANS1*/*LAR1*/*ANR1::LUC*, LUC activities were dramatically enhanced and were considerably higher (3- to 50-fold) than NtMYB330, NtAn1b or the reporter only controls (Figure 6). Taken together, these results demonstrated that NtAn1b is an essential co-activator of NtMYB330, and the formation of NtMYB330-NtAn1b complex is required for NtMYB330 to achieve full functionality in regulating PA biosynthetic genes.

### NtMYB330 Regulates Seed Coat Proanthocyanidins and Affects Seed Germination

**FIGURE 7:**
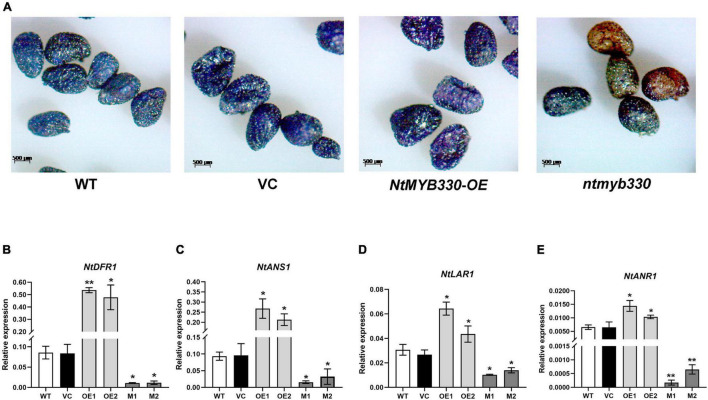
NtMYB330 regulates PA biosynthesis in tobacco seeds. **(A)** DMACA staining of PAs accumulated in the seed coats of WT, VC, *NtMYB330-OE* and *ntmyb330* mutant plants. Scale bar = 500 μm. Relative expression levels of **(B)** *NtDFR1*, **(C)** *NtANS1*, **(D)** *NtLAR1* and **(E)** *NtANR1* in the seeds of WT, VC, *NtMYB330-OE* and *ntmyb330* mutant plants. Data are the mean of three replicates with error bars indicating ± SD. Asterisks indicate statistically significant differences from WT according to paired *t*-test (*P < 0.05; **P < 0.01).

**FIGURE 8:**
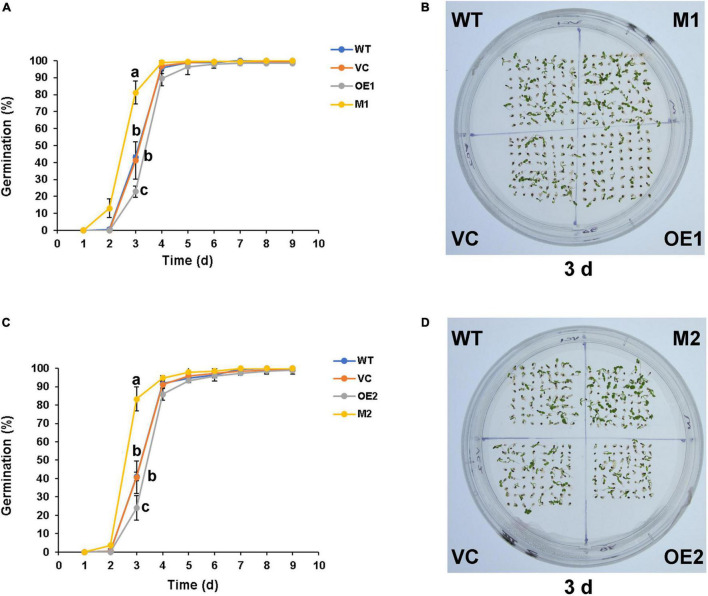
Seed germination phenotypes of WT, VC, *NtMYB330-OE* lines and *ntmyb330* mutant plants. **(A,C)** Seed germination percentages of WT, VC, *NtMYB330-OE* lines (OE1, OE2) and *ntmyb330* mutant plants (M1, M2) in nine consecutive days. Values are means of three independent replicates. Error bars denote standard deviations. Different letters indicate statistically significant differences at *P* < 0.05 according to Tukey’s HSD test. **(B,D)** Seedlings of WT, VC, *NtMYB330-OE* lines (OE1, OE2) and *ntmyb330* mutants (M1 and M2) grown on 1/2 MS medium for 3 days. The entire experiment was repeated three times, representative data from one experimental replicate presented here.

The seed coat colors of different lines were not significantly different before staining (Supplementary Figure 6). Following DMACA staining, seed coats of wild-type and vector control were in grayish blue (Figure 7A). Seeds of *NtMYB330-OE* lines exhibited dark blue color, while *ntmyb330* mutants had brown and/or chartreuse seed coats. *NtDFR1*, *NtANS1*, *NtLAR1*, and *NtANR1* in seeds were downregulated in *ntmyb330* mutants while upregulated in *NtMYB330-OE* lines compared to the wild-type (Figures 7B–E). Consistent with the PA regulation in flowers, these results suggested that NtMYB330 modulated LBG expressions to affect PA biosynthesis in seeds, leading to enhanced PA accumulations in *NtMYB330-OE* seed coats while decreased PA concentrations in *ntmyb330* seed coats. Seed coat PAs are positively associated with seed dormant state and affect seed germination (Jia L. et al., 2012). In this study, mature seeds of *ntmyb330* mutants germinated faster and the germination percentages of *ntmyb330* mutants at the third day were significantly higher than the wild-type and vector controls (Figure 8). In contrast, the seed germination percentages of *NtMYB330-OE* lines were significantly lower at the third day. It is possible that enhanced seed coat PAs increase the thickness while reduce the water permeability of seed coats, leading to inhibition of germination, and vice versa.

**FIGURE 9:**
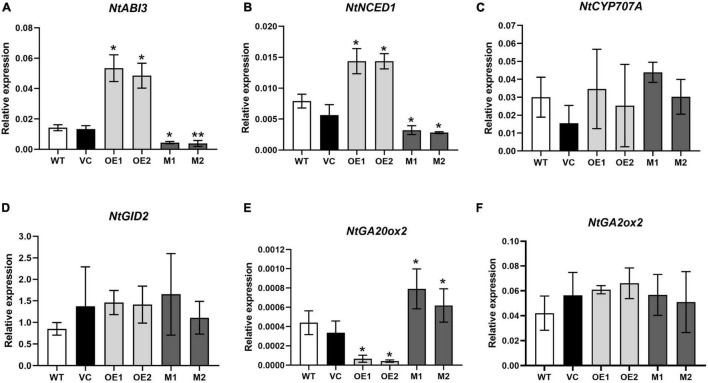
Quantitative analyses of transcript levels of the genes involved in homeostasis and signal transduction of ABA/GA in tobacco seeds. Relative expression levels of **(A)** *NtABI3* (ABA signaling-related gene), **(B)** *NtNCED1* (gene involved in ABA biosynthesis), **(C)** *NtCYP707A* (gene involved in ABA catabolism), **(D)** *NtGID2* (GA signaling-related gene), **(E)** *NtGA20ox2* (gene involved in GA biosynthesis), and **(F)** *NtGA2ox2* (gene involved in GA catabolism) in the seeds of WT, VC, *NtMYB330-OE* lines and *ntmyb330* mutant plants. Data are the mean of three replicates with error bars indicating ± SD. Asterisks indicate statistically significant differences from WT according to paired *t*-test (*P < 0.05; **P < 0.01).

In tomato, a flavonoid-regulating WDR protein SlAn11 was found to affect seed germination by adjusting seed coat PA concentrations and/or directly modulating the genes involved in ABA-GA crosstalk (Gao et al., 2018). To determine whether or not NtMYB330 affects seed germination by regulating ABA/GA signaling-related genes, the transcript levels of genes involved in ABA/GA biosynthesis, catabolism and signaling pathways were quantified (Figure 9). The expressions of *NtABI3* (ABA signaling-related gene) and *NtNCED1* (key gene involved in ABA biosynthesis) were increased in the seeds of *NtMYB330-OE* lines while decreased in the seeds of *ntmyb330* mutants compared with the wild-type (Figures 9A,B). The transcript levels of *NtGA20ox2* (key gene involved in GA biosynthesis) were significantly decreased in *NtMYB330-OE* seeds while increased in *ntmyb330* mutant seeds (Figure 9E). The expressions of *NtCYP707A* (key gene involved in ABA catabolism), *NtGID2* (GA signaling-related gene) and *NtGA2ox2* (gene triggers GA inactivation) were not significantly changed in either *NtMYB330-OE* or *ntmyb330* mutant seeds compared to wild-type (Figures 9C,D,F). These results showed that *NtABI3* was positively regulated by NtMYB330, and disruption of *NtMYB330* inhibited ABA biosynthesis while promoted GA biosynthesis, suggesting a regulatory role of NtMYB330 in affecting seed germination through direct effect on ABA-GA crosstalk.

## Discussion

### NtMYB330 Affects Proanthocyanidin Biosynthesis Through Regulating Late Biosynthetic Genes of Flavonoid Pathway

Proanthocyanidin biosynthesis in higher plants has been studied extensively and many MYB factors that specifically regulate PA biosynthetic genes have been cloned and functionally analyzed (Dixon and Sarnala, 2020). Tobacco has been mostly used as a model system to analyze the gene function of exogenous PA-specific MYBs. However, its own PA-regulating MYB has not been identified. In this study, NtMYB330 protein shares significant amino acid homologies with a number of AtTT2-like MYBs and its protein C-terminal domain harbors the signature motif known to be associated with PA-clade 2 regulators (Hichri et al., 2011a). *NtMYB330* has two introns (Supplementary Figure 1B) and mainly expressed in tobacco flowers and seeds (Figure 2B). The functional specificity of NtMYB330 was verified by generating *NtMYB330* overexpression lines and knockout mutants. The floral and seed coat PA concentrations were significantly higher in *NtMYB330-OE* lines while lower in *ntmyb330* mutants, compared with the controls (Figures 4B, 7A). These results demonstrated that NtMYB330 is a PA-clade 2 MYB regulator, specifically controlling the PA biosynthesis in tobacco flowers and seeds.

PA-related LBGs (*NtDFRs*, *NtANSs*, *NtLARs*, and *NtANRs*) were upregulated and downregulated in the flowers of *NtMYB330-OE* lines and *ntmyb330* mutants, respectively (Figures 3C–J), while EBGs including *NtCHSs*, *NtCHIs NtF3Hs* and *NtFLSs*, commonly shared by flavonol, anthocyanin and PA pathways, were not significantly changed compared with the wild-type (Supplementary Figures 2A,B). PA-regulating MYB factors, such as AtTT2 from *Arabidopsis*, FaMYB9/FaMYB11 from strawberry, MdMYB23 from apple and TaMYB14 from *Trifolium arvense*, were also found to specifically target LBGs of the PA branch (Nesi et al., 2001; Hancock et al., 2012; Schaart et al., 2013; An et al., 2018). At the meanwhile, there are PA regulators that can modulate both EBGs and LBGs of the flavonoid pathway, such as VvMYBPA1 from grape, MtPAR from *Medicago truncatula*, PtrMYB115 and PtrMYB134 from poplar (Bogs et al., 2007; Verdier et al., 2012; James et al., 2017; Wang et al., 2017). Given the sequence and functional conservation of these PA-regulating MYB factors, the species-specific regulatory differences might be attributed to the promoter divergence in structural genes rather than divergence of the regulators (Quattrocchio et al., 1998). Some PA-specific MYBs can directly regulate their interacting bHLH partners. For example, TT2-like GtMYB3 from gentian flower petals can control TT8-like *GtbHLH* expression (Nakatsuka et al., 2008). MYB-bHLH dimers were also found to regulate the bHLH factors in Arabidopsis and grape (Baudry et al., 2006; Hichri et al., 2010). However, the expressions of *NtAn1b* and *NtAn11-1* genes were not altered by NtMYB330 (Supplementary Figures 2C,D). Our results indicated that NtMYB330, as a PA-specific transcription factor, has a conserved role in regulating PA biosynthesis in tobacco, while its specific regulation of LBGs and its inability to regulate other members of the MBW complex indicate the diversity of underlying mechanisms in PA regulation in higher plants.

### NtMYB330-Dependent Complex Is Involved in Regulating Proanthocyanidin Pathway in Tobacco

In higher plants, PA biosynthesis is transcriptionally regulated by the ternary MBW protein complex consisting of one R2R3-MYB protein like Arabidopsis TT2, one bHLH protein like Arabidopsis TRANSPARENT TESTA 8 (TT8) and one WDR protein like Arabidopsis TRANSPARENT TESTA GLABRA 1 (TTG1) (Baudry et al., 2004). NtAn1b (bHLH) and NtAn11-1 (WDR) were isolated from tobacco flowers and can interact with the anthocyanin-specific regulator NtAn2 to form a regulatory complex and affect anthocyanin accumulations in tobacco flowers (Pattanaik et al., 2010). In this study, although NtMYB330 alone was shown to activate *NtDFR1*, *NtLAR1*, and *NtANR1* in the dual luciferase assay (Figures 6A,C,D), possibly due to the presence of other NtMYB330-interacting bHLH partners in *N. benthamiana* leaf cells, NtMYB330-NtAn1b complex was required for NtMYB330 to achieve full functionality in activating these PA branch genes (Figure 6). Y2H and BiFC assays indicated that NtMYB330 can form regulatory complexes with flavonoid-related bHLH regulator (NtAn1b) and WDR protein (NtAn11-1) (Figure 5) to control PA biosynthesis. This type of regulatory mechanism is consistent with orthologous MYB factors characterized in strawberry, kiwifruit, *Anthurium andraeanum*, *Freesia hybrida* and poplar (Schaart et al., 2013; Wang et al., 2017, 2019; Li Y. et al., 2018; Li et al., 2019). However, VvMYBPAR, although clustered with NtMYB330 in the same branch (Figure 1B), can directly activate the PA-specific genes *VvANR*, *VvLAR1*, and *VvLAR2* without interacting with bHLH or WDR partners (Koyama et al., 2014). These results indicated that NtMYB330-dependent complex is involved in regulating floral and seed coat PA biosynthesis in tobacco. It needs further study to determine whether there is a PA-regulating MYB factor that is independent of co-activators in tobacco.

In higher plants, it is the MYB factor that specify the biological function of the MBW regulatory complex. In Arabidopsis, anthocyanin-specific regulator AtPAP1/2 and PA-specific regulator AtTT2 can interact with the same bHLH (AtTT8) and WDR protein (AtTTG1) to form functionally different transcription complexes AtPAP1/2-AtTT8-AtTTG1 and AtTT2-AtTT8-AtTTG1, regulating anthocyanin and PA biosynthesis, respectively (Feller et al., 2011). In tobacco, mutation of *NtAn1* genes (*NtAn1b* and *NtAn1b*) resulted in defects in PA deposition in seed coats and reductions in anthocyanin accumulation in flowers (Tian et al., 2020), suggesting the non-specificity of NtAn1b, which can participate in the regulations of different branches of the flavonoid pathway. In previous study, NtAn1b and NtAn11-1 were characterized as the co-activators of NtAn2 and involved in regulating anthocyanin biosynthesis in tobacco (Bai et al., 2011). Our data showed that NtMYB330 can form the regulatory complex with NtAn1b and NtAn11-1 to specifically regulate PA branch, therefore, NtMYB330 determines the regulatory specificity of the MBW complex.

### NtMYB330 Regulates Proanthocyanidin Biosynthesis in Seed Coats and Seed Germination

Mutation of *NtMYB330* promoted seed germination, whereas overexpression of *NtMYB330* inhibited seed germination under normal conditions (Figure 8). Influence on seed germination by NtMYB330 is likely to be associated with different deposition of PAs in seed coats (Figure 7A), in which higher PA accumulations in *NtMYB330*-*OE* seeds can result in more intense coat pigmentation, less water permeability of seeds and more dormant state, and vice versa (MacGregor et al., 2015). Arrested seed germination has been widely observed in the seeds that are high in PAs (Jia L.G. et al., 2012; Freixas Coutin et al., 2017; Gao et al., 2018), in which PA concentrations could affect the thickness of seed coats and lead to effect on germination.

The dynamic balance of ABA and GA is crucial for seed germination, in which ABA acts as an inhibitor of seed germination whereas GA functions as an important signal to break seed dormancy and promote germination (Finkelstein et al., 2008). *NCED* (encoding 9-cis-epoxycarotenoid dioxygenase) and *CYP707A* (encoding ABA 8-hydroxylases) are key genes involved in ABA biosynthesis and catabolism, respectively (Footitt et al., 2011). *GA20ox* (encoding GA 20-oxidase) are key genes in GA biosynthesis, whereas *GA2ox* (encoding GA 2-oxidase) triggers GA inactivation (Li Z. et al., 2018). *ABI3* (ABA insensitive 3) and *GID* (gibberellin insensitive dwarf protein) are genes encoding the receptors of ABA and GA signaling pathways, respectively (Griffiths et al., 2006). Interestingly, our data revealed that NtMYB330 can regulate the expressions of *NtNCED1* and *NtGA20ox2*, the key genes involved in ABA and GA biosynthesis, respectively, and the expression of ABA-signaling related gene *NtABI3* (Figure 9). In tomato, the PA-related WD40 transcription factor SlAN11 was also found to affect seed germination by positively regulating *SlABI3* and *SlABI5* (Gao et al., 2018). In addition, the inhibition of seed germination by PAs was found to be associated with the enhancement of ABA *de novo* synthesis (Jia L. et al., 2012). While, a putative GA-regulated protein gene was upregulated in PA deficient non-darkening cranberry beans (Freixas Coutin et al., 2017). Therefore, in addition to coat-imposed dormancy, the possibility remains that NtMYB330 affects seed germination by regulating the ABA/GA biosynthesis and modulating the ABA-GA crosstalk. The regulatory mechanism of NtMYB330 on ABA/GA biosynthesis during seed germination needs further study.

In this study, we characterized a PA-specific MYB regulator in tobacco, which regulates PA biosynthesis in tobacco flowers via formation of the MBW complex and affects seed germination through modulating PA concentrations and ABA/GA signaling in tobacco seeds. The generated *ntmyb330* mutant can serve as a valuable mutant platform for functional complementation tests of PA-specific MYB regulators isolated from other plant species. Moreover, it is a proper genetic resource to further understand the important cues (such as temperatures, phytohormones, small molecule signals and so on) (MacGregor et al., 2015; Lv et al., 2021) that might determine the depth of tobacco seed dormancy.

## Data Availability Statement

The original contributions presented in the study are included in the article/Supplementary material, further inquiries can be directed to the corresponding author/s.

## Author Contributions

LZ and ZS conceived the project and designed the study. LZ wrote the manuscript. BW, YG, and JS performed the experiments. XS, XC, and YZ contributed to data acquisition. YL supervised the research and reviewed the manuscript. All authors contributed to the article and approved the submitted version.

## Conflict of Interest

The authors declare that the research was conducted in the absence of any commercial or financial relationships that could be construed as a potential conflict of interest.

## Publisher’s Note

All claims expressed in this article are solely those of the authors and do not necessarily represent those of their affiliated organizations, or those of the publisher, the editors and the reviewers. Any product that may be evaluated in this article, or claim that may be made by its manufacturer, is not guaranteed or endorsed by the publisher.

## Footnotes

1
https://solgenomics.net/organism/Nicotiana_tabacum

## References

[B1] Abrahams S. Tanner G. J. Larkin P. J. Ashton A. R. (2002). Identification and biochemical characterization of mutants in the proanthocyanidin pathway in *Arabidopsis*. *Plant Physiol.* 130 561–576. 10.1104/pp.006189 12376625 PMC166587

[B2] Akagi T. Ikegami A. Yonemori K. (2010). DkMyb2 wound-induced transcription factor of persimmon (*Diospyros kaki* Thunb.), contributes to proanthocyanidin regulation. *Planta* 232 1045–1059. 10.1007/s00425-010-1241-7 20690029

[B3] An J. P. Li R. Qu F. J. You C. X. Wang X. F. Hao Y. J. (2018). R2R3-MYB transcription factor MdMYB23 is involved in the cold tolerance and proanthocyanidin accumulation in apple. *Plant J.* 96 562–577. 10.1111/tpj.14050 30054966

[B4] Appelhagen I. Lu G. H. Huep G. Schmelzer E. Weisshaar B. Sagasser M. (2011). Transparent TESTA1 interacts with R2R3-MYB factors and affects early and late steps of flavonoid biosynthesis in the endothelium of *Arabidopsis thaliana* seeds. *Plant J.* 67 406–419. 10.1111/j.1365-313X.2011.04603.x 21477081

[B5] Bai Y. Pattanaik S. Patra B. Werkman J. R. Xie C. H. Yuan L. (2011). Flavonoid-related basic helix-loop-helix regulators, NtAn1a and NtAn1b, of tobacco have originated from two ancestors and are functionally active. *Planta* 234 363–375. 10.1007/s00425-011-1407-y 21484270

[B6] Baudry A. Caboche M. Lepiniec L. (2006). TT8 controls its own expression in a feedback regulation involving TTG1 and homologous MYB and bHLH factors, allowing a strong and cell-specific accumulation of flavonoids in *Arabidopsis thaliana*. *Plant J.* 46 768–779. 10.1111/j.1365-313X.2006.02733.x 16709193

[B7] Baudry A. Heim M. A. Dubreucq B. Caboche M. Weisshaar B. Lepiniec L. (2004). TT2, TT8, and TTG1 synergistically specify the expression of BANYULS and proanthocyanidin biosynthesis in *Arabidopsis thaliana*. *Plant J.* 39 366–380. 10.1111/j.1365-313X.2004.02138.x 15255866

[B8] Baxter I. R. Young J. C. Armstrong G. Foster N. Bogenschutz N. Cordova T. (2005). A plasma membrane H+-ATPase is required for the formation of proanthocyanidins in the seed coat endothelium of *Arabidopsis thaliana*. *Proc. Natl. Acad. Sci. U.S.A.* 102 2649–2654. 10.1073/pnas.0406377102 15695592 PMC548969

[B9] Bogs J. Downey M. O. Harvey J. S. Ashton A. R. Tanner G. J. Robinson S. P. (2005). Proanthocyanidin synthesis and expression of genes encoding leucoanthocyanidin reductase and anthocyanidin reductase in developing grape berries and grapevine leaves. *Plant Physiol.* 139 652–663. 10.1104/pp.105.064238 16169968 PMC1255985

[B10] Bogs J. Jaffe F. W. Takos A. M. Walker A. R. Robinson S. P. (2007). The grapevine transcription factor VvMYBPA1 regulates proanthocyanidin synthesis during fruit development. *Plant Physiol.* 143 1347–1361. 10.1104/pp.106.093203 17208963 PMC1820911

[B11] Dixon R. A. Sarnala S. (2020). Proanthocyanidin biosynthesis-a matter of protection. *Plant Physiol.* 184 579–591. 10.1104/pp.20.00973 32817234 PMC7536678

[B12] Dixon R. A. Xie D. Y. Sharma S. B. (2005). Proanthocyanidins–a final frontier in flavonoid research? *New Phytol.* 165 9–28. 10.1111/j.1469-8137.2004.01217.x 15720617

[B13] Feller A. Machemer K. Braun E. L. Grotewold E. (2011). Evolutionary and comparative analysis of MYB and bHLH plant transcription factors. *Plant J.* 66 94–116. 10.1111/j.1365-313X.2010.04459.x 21443626

[B14] Finkelstein R. Reeves W. Ariizumi T. Steber C. (2008). Molecular aspects of seed dormancy. *Annu. Rev. Plant Biol.* 59 387–415. 10.1146/annurev.arplant.59.032607.092740 18257711

[B15] Footitt S. Douterelo-Soler I. Clay H. Finch-Savage W. E. (2011). Dormancy cycling in Arabidopsis seeds is controlled by seasonally distinct hormone-signaling pathways. *Proc. Natl. Acad. Sci. U.S.A.* 108 20236–20241. 10.1073/pnas.1116325108 22128331 PMC3250134

[B16] Freixas Coutin J. A. Munholland S. Silva A. Subedi S. Lukens L. Crosby W. L. (2017). Proanthocyanidin accumulation and transcriptional responses in the seed coat of cranberry beans (*Phaseolus vulgaris* L.) with different susceptibility to postharvest darkening. *BMC Plant Biol.* 17:89. 10.1186/s12870-017-1037-z 28545577 PMC5445279

[B17] Gao Y. Liu J. Chen Y. Tang H. Wang Y. He Y. (2018). Tomato SlAN11 regulates flavonoid biosynthesis and seed dormancy by interaction with bHLH proteins but not with MYB proteins. *Hortic. Res.* 5:27. 10.1038/s41438-018-0032-3 29872532 PMC5981465

[B18] Griffiths J. Murase K. Rieu I. Zentella R. Zhang Z. L. Powers S. J. (2006). Genetic characterization and functional analysis of the GID1 gibberellin receptors in *Arabidopsis*. *Plant Cell* 18 3399–3414. 10.1105/tpc.106.047415 17194763 PMC1785415

[B19] Hancock K. R. Collette V. Fraser K. Greig M. Xue H. Richardson K. (2012). Expression of the R2R3-MYB transcription factor TaMYB14 from Trifolium arvense activates proanthocyanidin biosynthesis in the legumes *Trifolium repens* and *Medicago sativa*. *Plant Physiol.* 159 1204–1220. 10.1104/pp.112.195420 22566493 PMC3387705

[B20] Hellens R. P. Allan A. C. Friel E. N. Bolitho K. Grafton K. Templeton M. D. (2005). Transient expression vectors for functional genomics, quantification of promoter activity and RNA silencing in plants. *Plant Methods* 1:13. 10.1186/1746-4811-1-13 16359558 PMC1334188

[B21] Heppel S. C. Jaffe F. W. Takos A. M. Schellmann S. Rausch T. Walker A. R. (2013). Identification of key amino acids for the evolution of promoter target specificity of anthocyanin and proanthocyanidin regulating MYB factors. *Plant Mol. Biol.* 82 457–471. 10.1007/s11103-013-0074-8 23689818

[B22] Hichri I. Barrieu F. Bogs J. Kappel C. Delrot S. Lauvergeat V. (2011a). Recent advances in the transcriptional regulation of the flavonoid biosynthetic pathway. *J. Exp. Bot.* 62 2465–2483. 10.1093/jxb/erq442 21278228

[B23] Hichri I. Deluc L. Barrieu F. Bogs J. Mahjoub A. Regad F. (2011b). A single amino acid change within the R2 domain of the VvMYB5b transcription factor modulates affinity for protein partners and target promoters selectivity. *BMC Plant Biol.* 11:117. 10.1186/1471-2229-11-117 21861899 PMC3240579

[B24] Hichri I. Heppel S. C. Pillet J. Leon C. Czemmel S. Delrot S. (2010). The basic helix-loop-helix transcription factor MYC1 is involved in the regulation of the flavonoid biosynthesis pathway in grapevine. *Mol. Plant* 3 509–523. 10.1093/mp/ssp118 20118183

[B25] James A. M. Ma D. Mellway R. Gesell A. Yoshida K. Walker V. (2017). Poplar MYB115 and MYB134 transcription factors regulate proanthocyanidin synthesis and structure. *Plant Physiol.* 174 154–171. 10.1104/pp.16.01962 28348066 PMC5411147

[B26] Jia L. Wu Q. Ye N. Liu R. Shi L. Xu W. (2012). Proanthocyanidins inhibit seed germination by maintaining a high level of abscisic acid in *Arabidopsis thaliana*. *J. Integr. Plant Biol.* 54 663–673. 10.1111/j.1744-7909.2012.01142.x 22765383

[B27] Jia L. G. Sheng Z. W. Xu W. F. Li Y. X. Liu Y. G. Xia Y. J. (2012). Modulation of anti-oxidation ability by proanthocyanidins during germination of *Arabidopsis thaliana* seeds. *Mol. Plant* 5 472–481. 10.1093/mp/ssr089 22115918

[B28] Jiao F. Zhao L. Wu X. Song Z. Li Y. (2020). Metabolome and transcriptome analyses of the molecular mechanisms of flower color mutation in tobacco. *BMC Genomics* 21:611. 10.1186/s12864-020-07028-5 32894038 PMC7487631

[B29] Karimi M. Inze D. Depicker A. (2002). GATEWAY vectors for *Agrobacterium*-mediated plant transformation. *Trends Plant Sci.* 7 193–195. 11992820 10.1016/s1360-1385(02)02251-3

[B30] Koyama K. Numata M. Nakajima I. Goto-Yamamoto N. Matsumura H. Tanaka N. (2014). Functional characterization of a new grapevine MYB transcription factor and regulation of proanthocyanidin biosynthesis in grapes. *J. Exp. Bot.* 65 4433–4449. 10.1093/jxb/eru213 24860184

[B31] Lepiniec L. Debeaujon I. Routaboul J. M. Baudry A. Pourcel L. Nesi N. (2006). Genetics and biochemistry of seed flavonoids. *Annu. Rev. Plant Biol.* 57 405–430. 10.1146/annurev.arplant.57.032905.105252 16669768

[B32] Li C. Qiu J. Huang S. Yin J. Yang G. (2019). AaMYB3 interacts with AabHLH1 to regulate proanthocyanidin accumulation in *Anthurium andraeanum* (Hort.)-another strategy to modulate pigmentation. *Hortic. Res.* 6:14. 10.1038/s41438-018-0102-6 30603098 PMC6312548

[B33] Li Y. Shan X. Zhou L. Gao R. Yang S. Wang S. (2018). The R2R3-MYB factor FhMYB5 From *Freesia hybrida* contributes to the regulation of anthocyanin and proanthocyanidin biosynthesis. *Front. Plant Sci.* 9:1935. 10.3389/fpls.2018.01935 30666265 PMC6330306

[B34] Li Y. G. Tanner G. Larkin P. (1996). The DMACA-HCl protocol and the thresholdproanthocyanidin content for bloat safety in forage legumes. *J. Sci. Food. Agric.* 70 89–101.

[B35] Li Z. Gao Y. Zhang Y. Lin C. Gong D. Guan Y. (2018). Reactive oxygen species and gibberellin acid mutual induction to regulate tobacco seed germination. *Front. Plant Sci.* 9:1279. 10.3389/fpls.2018.01279 30356911 PMC6190896

[B36] Lv Y. Pan J. Wang H. Reiter R. J. Li X. Mou Z. (2021). Melatonin inhibits seed germination by crosstalk with abscisic acid, gibberellin, and auxin in *Arabidopsis*. *J. Pineal Res.* 70:e12736. 10.1111/jpi.12736 33811388

[B37] MacGregor D. R. Kendall S. L. Florance H. Fedi F. Moore K. Paszkiewicz K. (2015). Seed production temperature regulation of primary dormancy occurs through control of seed coat phenylpropanoid metabolism. *New Phytol.* 205 642–652. 10.1111/nph.13090 25412428

[B38] Marles M. A. Ray H. Gruber M. Y. (2003). New perspectives on proanthocyanidin biochemistry and molecular regulation. *Phytochemistry* 64 367–383. 10.1016/s0031-9422(03)00377-7 12943753

[B39] Nakatsuka T. Haruta K. S. Pitaksutheepong C. Abe Y. Kakizaki Y. Yamamoto K. (2008). Identification and characterization of R2R3-MYB and bHLH transcription factors regulating anthocyanin biosynthesis in gentian flowers. *Plant Cell Physiol.* 49 1818–1829. 10.1093/pcp/pcn163 18974195

[B40] Nesi N. Jond C. Debeaujon I. Caboche M. Lepiniec L. (2001). The *Arabidopsis* TT2 gene encodes an R2R3 MYB domain protein that acts as a key determinant for proanthocyanidin accumulation in developing seed. *Plant Cell* 13 2099–2114. 11549766 10.1105/TPC.010098 PMC139454

[B41] Palmgren G. Mattson O. Okkels F. T. (1993). Treatment of *Agrobacterium* or leaf disks with 5-azacytidine increases transgene expression in tobacco. *Plant Mol. Biol.* 21 429–435. 10.1007/BF00028801 7680239

[B42] Park K. I. Ishikawa N. Morita Y. Choi J. D. Hoshino A. Iida S. (2007). A bHLH regulatory gene in the common morning glory, *Ipomoea purpurea*, controls anthocyanin biosynthesis in flowers, proanthocyanidin and phytomelanin pigmentation in seeds, and seed trichome formation. *Plant J.* 49 641–654. 10.1111/j.1365-313X.2006.02988.x 17270013

[B43] Passeri V. Martens S. Carvalho E. Bianchet C. Damiani F. Paolocci F. (2017). The R2R3MYB VvMYBPA1 from grape reprograms the phenylpropanoid pathway in tobacco flowers. *Planta* 246 185–199. 10.1007/s00425-017-2667-y 28299441

[B44] Pattanaik S. Kong Q. Zaitlin D. Werkman J. R. Xie C. H. Patra B. (2010). Isolation and functional characterization of a floral tissue-specific R2R3 MYB regulator from tobacco. *Planta* 231 1061–1076. 10.1007/s00425-010-1108-y 20157728

[B45] Quattrocchio F. Wing J. F. van der Woude K. Mol J. N. Koes R. (1998). Analysis of bHLH and MYB domain proteins: species-specific regulatory differences are caused by divergent evolution of target anthocyanin genes. *Plant J.* 13 475–488. 10.1046/j.1365-313x.1998.00046.x 9680994

[B46] Ramsay N. A. Glover B. J. (2005). MYB-bHLH-WD40 protein complex and the evolution of cellular diversity. *Trends Plant Sci.* 10 63–70. 10.1016/j.tplants.2004.12.011 15708343

[B47] Schaart J. G. Dubos C. Romero De La Fuente I. van Houwelingen A. M. de Vos R. C. Jonker H. H. (2013). Identification and characterization of MYB-bHLH-WD40 regulatory complexes controlling proanthocyanidin biosynthesis in strawberry (*Fragaria x ananassa*) fruits. *New Phytol.* 197 454–467. 10.1111/nph.12017 23157553

[B48] Terrier N. Torregrosa L. Ageorges A. Vialet S. Verries C. Cheynier V. (2009). Ectopic expression of VvMybPA2 promotes proanthocyanidin biosynthesis in grapevine and suggests additional targets in the pathway. *Plant Physiol.* 149 1028–1041. 10.1104/pp.108.131862 19098092 PMC2633825

[B49] Tian Y. Liu X. Fan C. Li T. Qin H. Li X. (2020). Enhancement of tobacco (*Nicotiana tabacum* L.) seed lipid content for biodiesel production by CRISPR-Cas9-mediated knockout of NtAn1. *Front. Plant Sci.* 11:599474. 10.3389/fpls.2020.599474 33552096 PMC7859101

[B50] Treutter D. (1989). Chemical reaction detection of catechins and proanthocyanidins with 4 dimethylaminocinnamaldehyde. *J. Chromatogr. A* 467 185–193.

[B51] Verdier J. Zhao J. Torres-Jerez I. Ge S. Liu C. He X. (2012). MtPAR MYB transcription factor acts as an on switch for proanthocyanidin biosynthesis in *Medicago truncatula*. *Proc. Natl. Acad. Sci. U.S.A.* 109 1766–1771. 10.1073/pnas.1120916109 22307644 PMC3277187

[B52] Wang L. Ran L. Hou Y. Tian Q. Li C. Liu R. (2017). The transcription factor MYB115 contributes to the regulation of proanthocyanidin biosynthesis and enhances fungal resistance in poplar. *New Phytol.* 215 351–367. 10.1111/nph.14569 28444797

[B53] Wang L. Tang W. Hu Y. Zhang Y. Sun J. Guo X. (2019). A MYB/bHLH complex regulates tissue-specific anthocyanin biosynthesis in the inner pericarp of red-centered kiwifruit *Actinidia chinensis* cv. *Hongyang. Plant J.* 99 359–378. 10.1111/tpj.14330 30912865

[B54] Xing H. L. Dong L. Wang Z. P. Zhang H. Y. Han C. Y. Liu B. (2014). A CRISPR/Cas9 toolkit for multiplex genome editing in plants. *BMC Plant Biol.* 14:327. 10.1186/s12870-014-0327-y 25432517 PMC4262988

[B55] Zhu Q. Sui S. Lei X. Yang Z. Lu K. Liu G. (2015). Ectopic expression of the coleus R2R3 MYB-Type proanthocyanidin regulator gene SsMYB3 alters the flower color in transgenic tobacco. *PLoS One* 10:e0139392. 10.1371/journal.pone.0139392 26448466 PMC4598174

