# Supplementary material for: R2R3-MYB Transcription Factor NtMYB330 Regulates Proanthocyanidin Biosynthesis and Seed Germination in Tobacco (Nicotiana tabacum L.)
Source: Front Plant Sci. 2022 Jan 17;12:819247. doi: 10.3389/fpls.2021.819247 (PMC8801704; doi:10.3389/fpls.2021.819247)
Supplement: Supplementary file 1 [file Data_Sheet_1.docx]

Supplementary Material

# Supplementary Figures

| **A** | **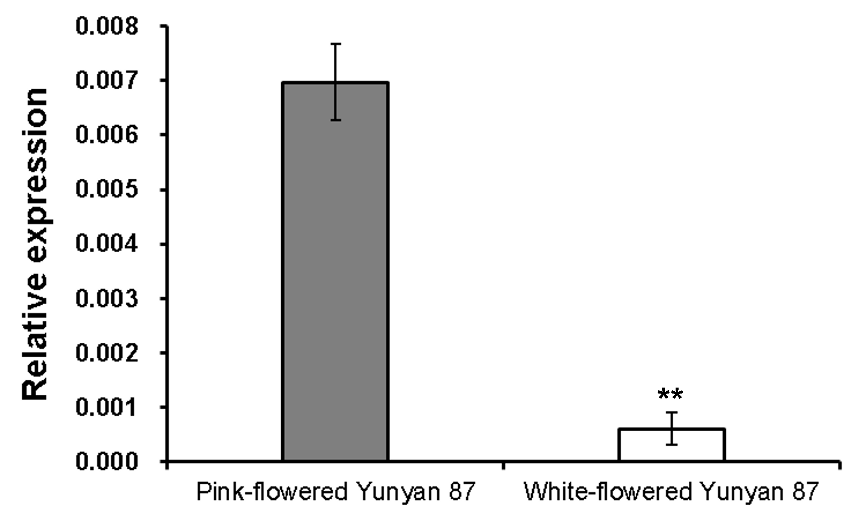** |
| --- | --- |
| **B** | **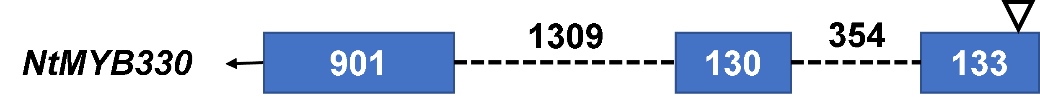** |

**Supplementary Figure 1**. **(A)** Relative expression levels of *NtMYB330* in the flowers of pink-flowered Yunyan 87 (wild-type) and white-flowered Yunyan 87 (mutant). Data are the mean of three replicates with error bars indicating ± SD. Asterisks indicate statistically significant differences from wild-type according to paired *t*-test (*, P < 0.05; **, P < 0.01). **(B)** Exon/intron structures of *NtMYB330* gene. Blue boxes and dash lines represent exons and introns, respectively, with numbers indicate the length. The inverted triangle indicates the PAM position for CRISPR/Cas9 system.

| **A** | **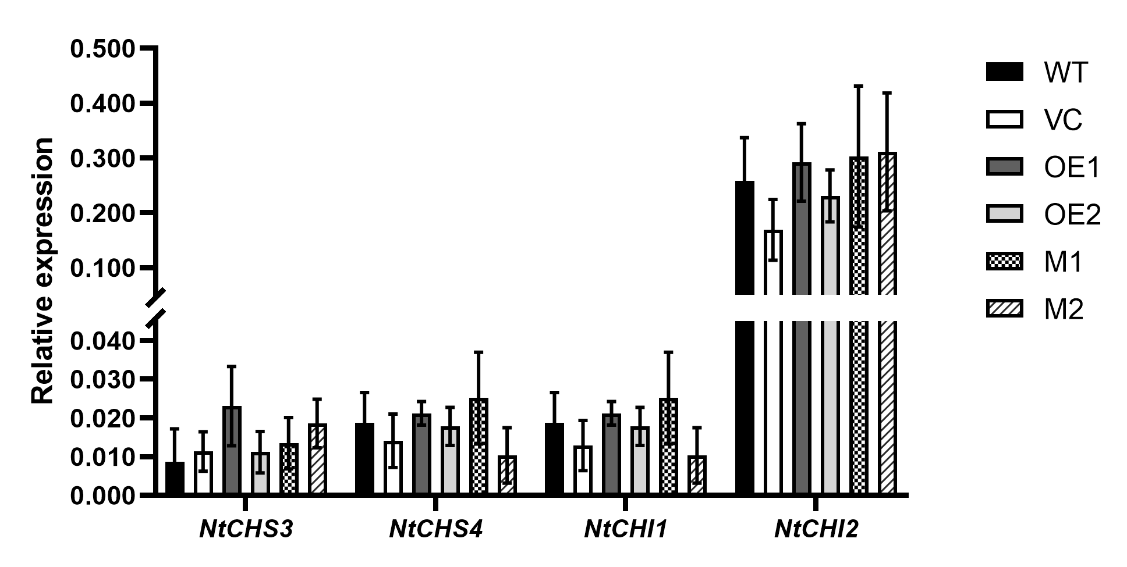** | | |
| --- | --- | --- | --- |
| **B** | **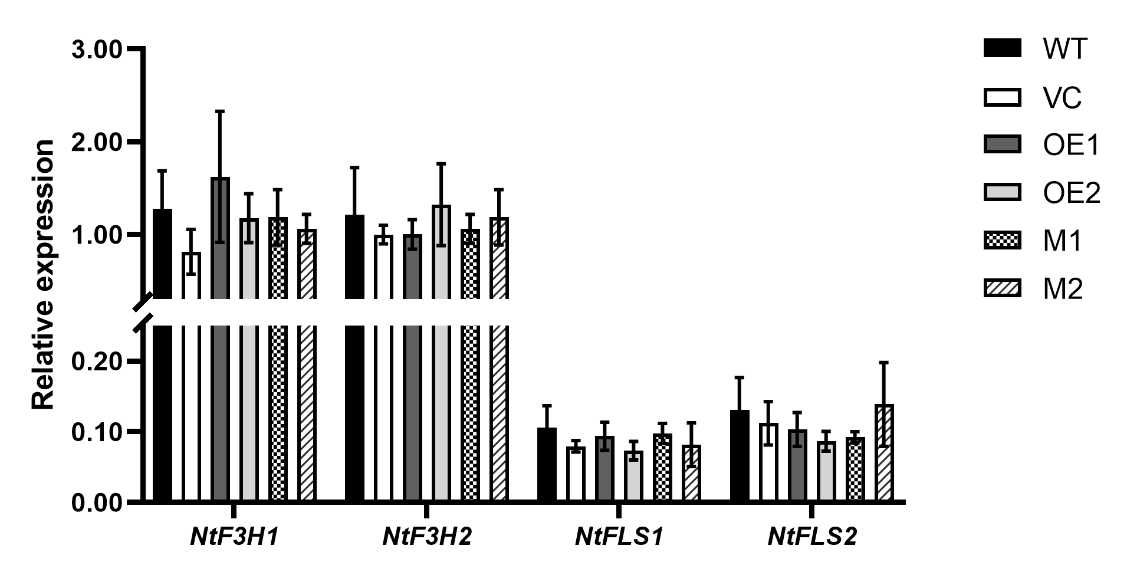** | | |
| **C** | **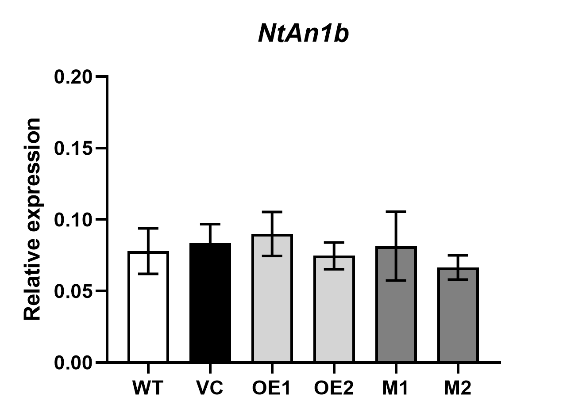** | **D** | **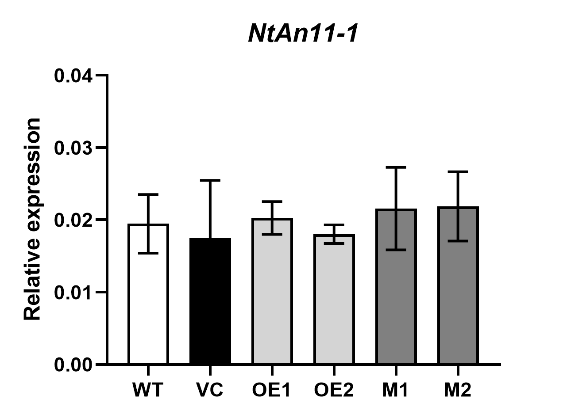** |

**Supplementary Figure 2.** Quantitative analyses of transcript levels of flavonoid-related EBGs, *NtAn1b* and *NtAn11-1* in tobacco flowers. Relative expression levels of **(A)** *NtCHS3*, *NtCHS4*, *NtCHI1*, *NtCHI2*, **(B)** *NtF3H1*, *NtF3H2*, *NtFLS1*, *NtFLS2*, **(C)** *NtAn1b*, and **(D)** *NtAn11-1* in the flower petals of WT, VC, *NtMYB330-OE* lines and *ntmyb330* mutant plants. Data are the mean of three replicates with error bars indicating $\pm$SD.

| **A** | **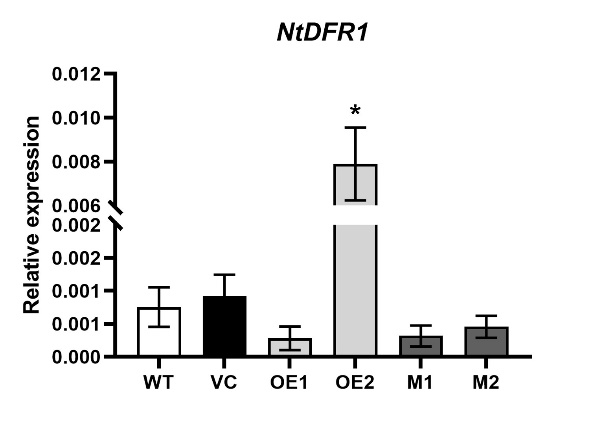** | **B** | 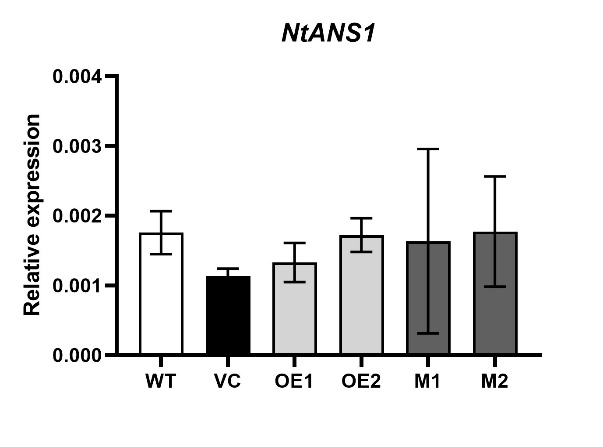 |
| --- | --- | --- | --- |
| **C** | **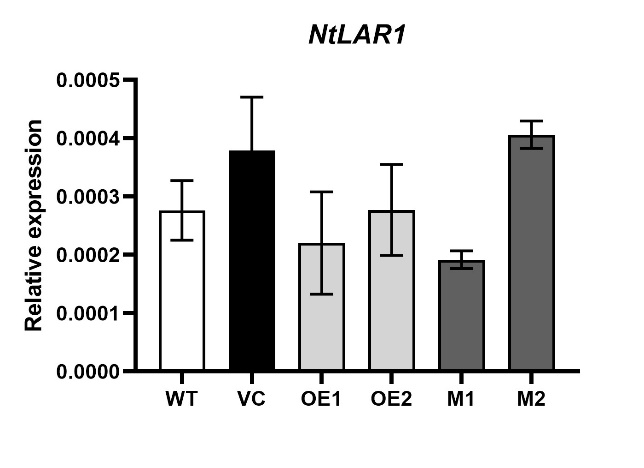** | **D** | 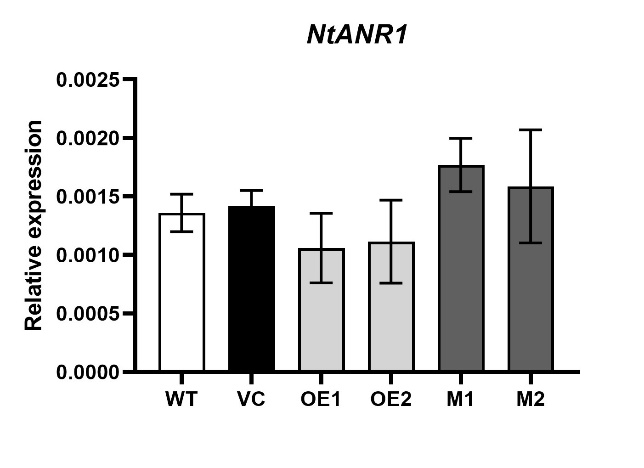 |

**Supplementary Figure 3.** Quantitative transcript analyses of LBGs in PA pathway in tobacco leaves. Analyses of expressions of **(A)** *NtDFR1*, **(B)** *NtANS1*, **(C)** *NtLAR1*and **(D)** *NtANR1* in the leaves of WT, VC, *NtMYB330-OE* lines and *ntmyb330* mutants. Data are the mean of three replicates with error bars indicating $\pm$SD. Asterisks indicate statistically significant differences from WT according to paired *t*-test (*, *P* < 0.05; **, *P* < 0.01).

| **A** | 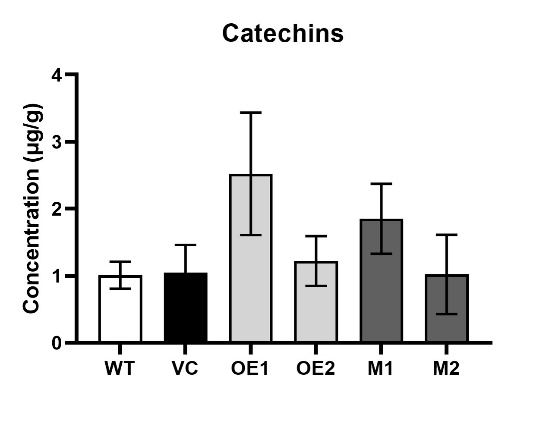 | **B** | **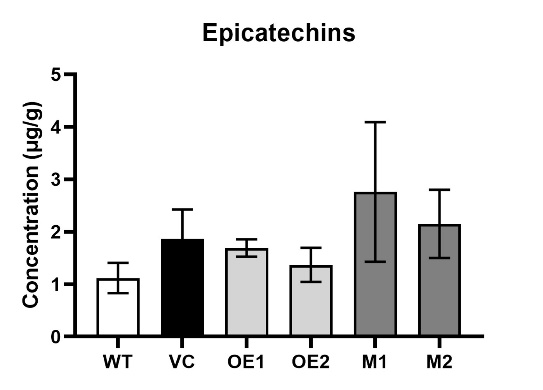** |
| --- | --- | --- | --- |

**Supplementary Figure 4.** Concentrations of **(A)** catechins and **(B)** epicatechins in the leaves of WT, VC, *NtMYB330-OE* lines and *ntmyb330* mutant plants. Data are the mean of three replicates with error bars indicating $\pm$SD.


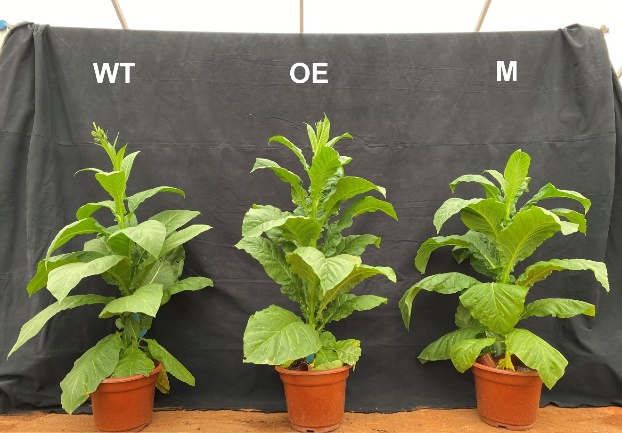


**Supplementary Figure 5.** WT, *NtMYB330-OE* and *ntmyb330* mutant plants at the flowering stage.

| **A** | **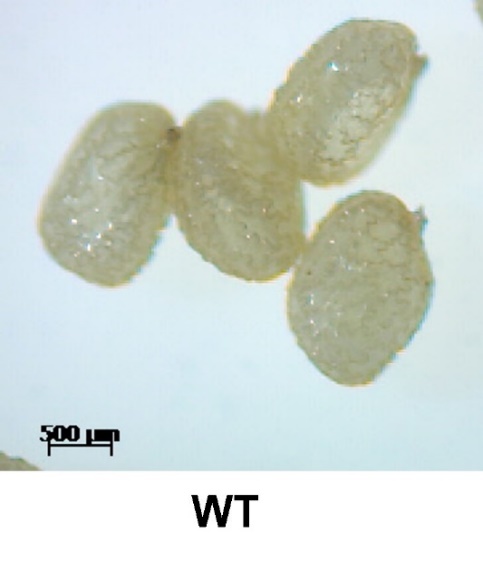** | **B** | **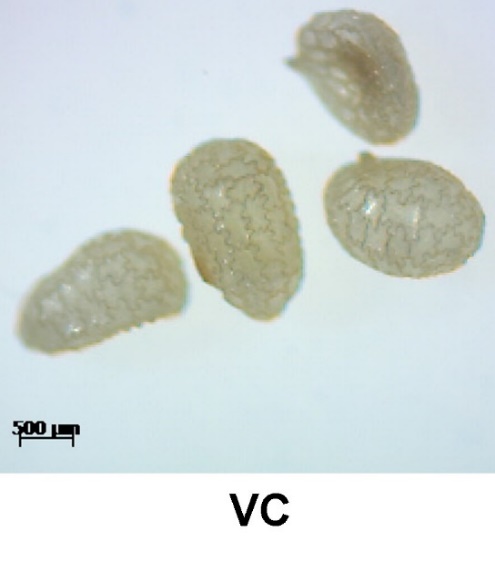** |
| --- | --- | --- | --- |
| **C** | **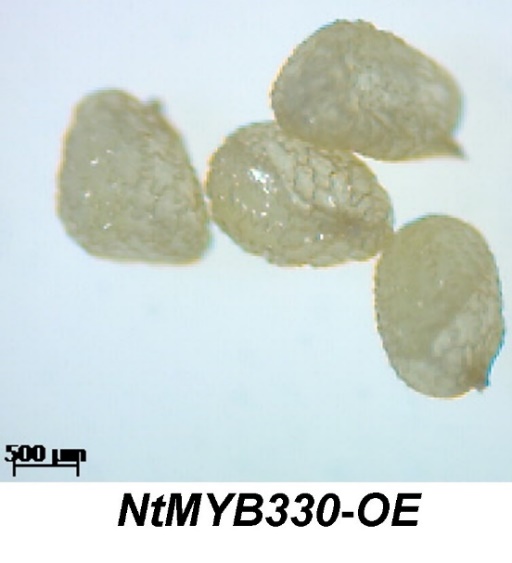** | **D** | **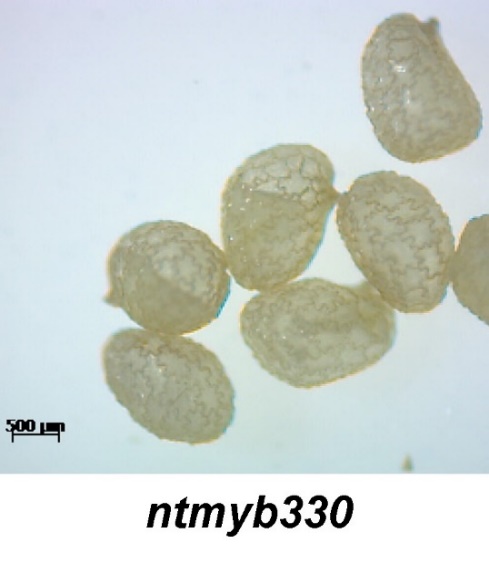** |

**Supplementary Figure 6.** Immature seeds of different lines without DMACA staining. **(A)** WT, **(B)** VC, **(C)** *NtMYB330-OE*, and **(D)** *ntmyb330*. Scale bar: 500 μm.

| **A** | 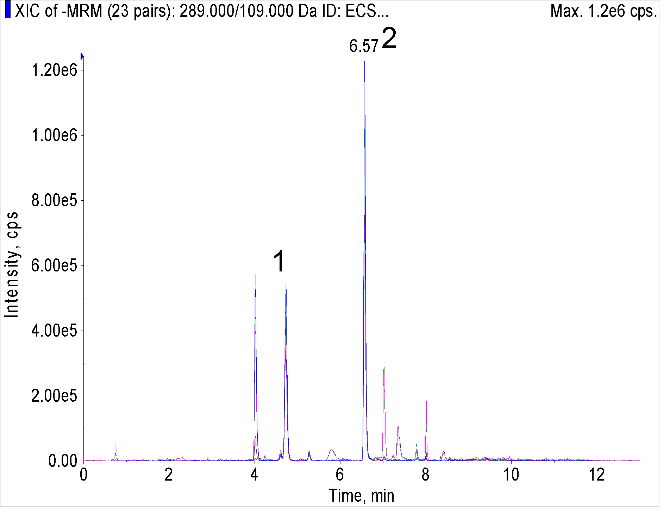 | **B** | 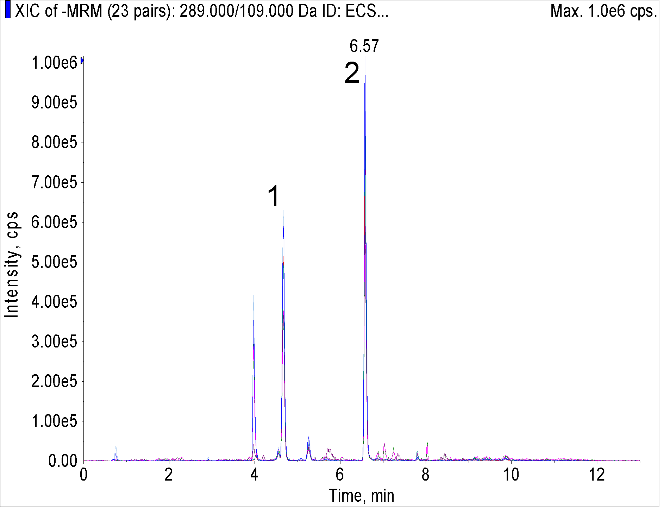 |
| --- | --- | --- | --- |
| **C** | 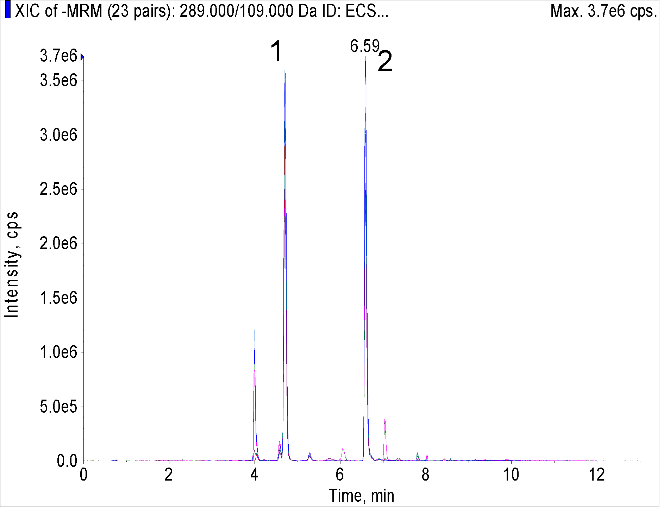 | **D** | 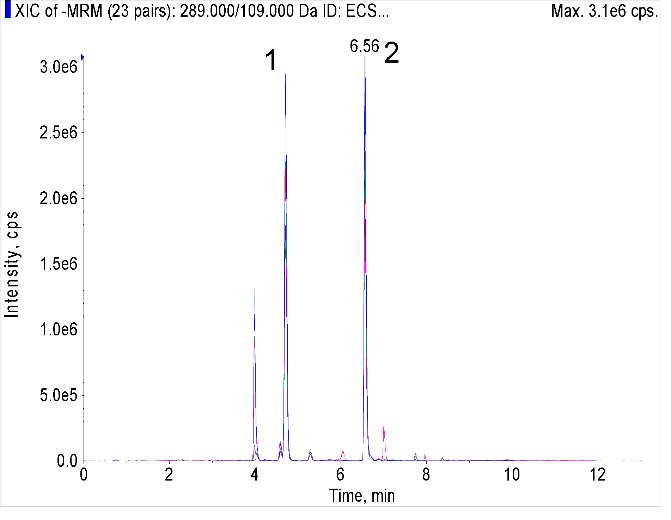 |
| **E** | 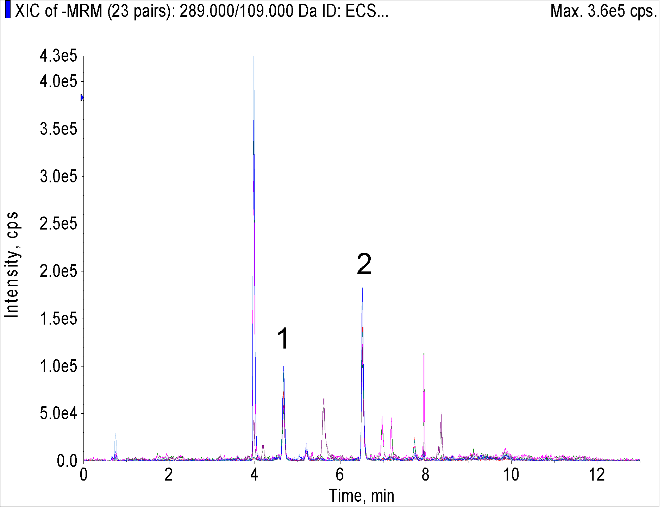 | **F** | 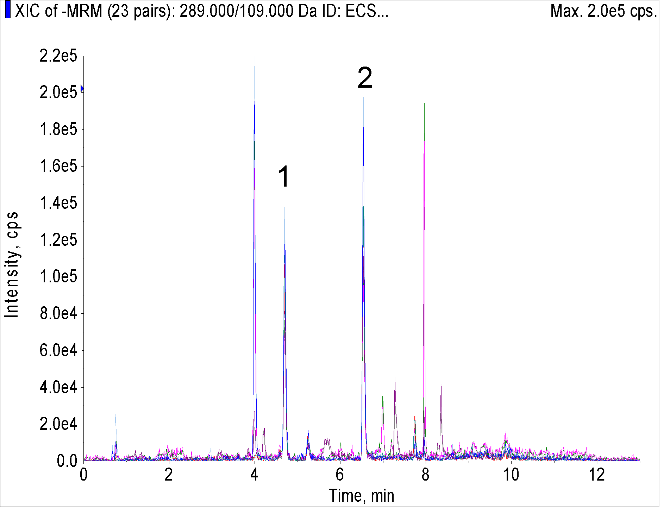 |
| **Supplementary Figure 7.** Analyses of catechins and epicatechins in the flower samples of **(A)** WT, **(B)** VC, **(C)** OE1, **(D)** OE2, **(E)** M1, **(F)** M2. Peak identities: (1) catechin; (2) epicatechin. Experiment was repeated three times, representative data from one experimental replicate presented here. | | | |
